# Exploring the therapeutic potential of recombinant bovine β-defensins for antimicrobial and anti-inflammatory functions in sepsis management

**DOI:** 10.1186/s13567-025-01601-0

**Published:** 2025-09-02

**Authors:** Cristina Saubi, Ricardo Baltà-Foix, Jose Vicente Carratalá, Francesc Fàbregas, Daniel Sandín, Marc Torrent, Elena Garcia-Fruitós, Anna Arís

**Affiliations:** 1 Ruminant Production, IRTA, Torre Marimon, 08140 Caldes de Montbui, Catalonia Spain; 2 Systems Biology of Infection Lab, Department of Biochemistry and Molecular Biology, Universitat Autònoma de Barcelona, 08193 Bellaterra, Barcelona Spain

**Keywords:** β-defensins, sepsis, LPS binding, LPS neutralisation, antimicrobial, *Escherichia coli*, recombinant proteins

## Abstract

β-defensins are multifunctional peptides of the host immune system involved in responses to infectious diseases. We investigated the potential of five recombinant proteins based on bovine β-defensins (bovine neutrophil β-defensins (BNBD) 1, 2, 3, and 4, and the tracheal antimicrobial peptide (TAP)) in functions relevant to sepsis such as antimicrobial activity, lipopolysaccharide (LPS) binding and neutralisation, and the stimulation of cytokine response in epithelial cells. These β-defensins were produced in *Lactococcus lactis* as fusion proteins. Antimicrobial activity was tested against *Escherichia coli*; LPS binding and neutralisation were assessed using a fluorescent probe displacement assay and by measuring tumour necrosis factor alpha (TNFα) levels in whole blood after an LPS challenge, respectively. Interleukin-8 (IL-8) levels were quantified to evaluate the epithelial immune response. All β-defensins exhibited different properties, suggesting they may have distinct mechanisms and functions in resolving infections. The recombinant BNBD4 showed potent antimicrobial activity against *E. coli*, whereas TAP was more notable for its ability to bind and neutralise LPS. These findings suggest that β-defensins, particularly BNBD4 and TAP, may be utilised to treat sepsis by targeting bacterial pathogens and modulating inflammatory responses.

**Supplementary Information:**

The online version contains supplementary material available at 10.1186/s13567-025-01601-0.

## Introduction

Β-defensins are a family of host defence peptides (HDPs), short, cationic, and amphipathic peptides from the innate immune system characterised by a conserved structure comprising a triple antiparallel β-sheet and three disulfide bonds. The bovine tracheal antimicrobial peptide (TAP) was the first β-defensin identified [1], followed by 13 β-defensins from bovine neutrophils, designated bovine neutrophil β-defensins (BNBD) 1–13 [2]. All exhibit antimicrobial activity against Gram-negative and Gram-positive bacteria, although to varying degrees [1, 2]. β-defensins are known to have chemotactic activity, to induce the production of cytokines and chemokines, to promote wound healing, and to bind and neutralise endotoxins [3–9]. However, these immunomodulatory activities have been poorly investigated in bovine β-defensins. Here, we specifically investigated the roles of TAP, bovine neutrophil β-defensin 1 (BNBD1), BNBD3, and BNBD4, as these HDPs have been shown in vitro and in vivo to be stimulated by immune challenges (e.g. endotoxins or bacteria) in the trachea [10], endometrium [11], salivary [12] and mammary glands [13–16], and ileum [17]. Moreover, in humans, transcription of β-defensin-2 in leukocytes of patients with severe sepsis is suppressed [18].

β-defensins can be chemically synthesised [19]. This approach yields high-purity products, but it has several limitations, including constraints on peptide length (typically suitable for peptides shorter than 35 amino acids), challenges in large-scale production, the use of toxic reagents with negative environmental impacts, and high production costs [20, 21]. A scalable and less expensive alternative is the recombinant production of β-defensins. Some bovine β-defensins have been successfully produced in previous studies: BNBD2, BNBD12, and LAP in *Escherichia coli* (*E. coli*) [22–24] and BNBD-5 in *Pichia pastoris* [25]. *E. coli* is the most commonly used cell factory due to its high expression levels and well-established methodologies, but the presence of lipopolysaccharides (LPS) necessitates additional purification. By contrast, *Lactococcus lactis* (*L. lactis*) is a Gram-positive bacterium generally recognised as safe (GRAS) and has also been employed to produce human and avian β-defensins [26, 27].

Due to their multiple functions, β-defensins may be valuable in treating sepsis, a life-threatening condition characterised by multi-organ dysfunction resulting from a dysregulated host response to infection [28, 29]. In cattle, sepsis is frequently caused by Gram-negative bacteria, particularly *E. coli* [30–33], and is common among critically ill calves, significantly increasing their risk of mortality [34]. Most antibiotic treatments are ineffective because antimicrobial resistance is common in bacterial isolates from calves with sepsis [34]. The outer membrane of Gram-negative bacteria contains LPS, endotoxins considered major mediators in sepsis [28, 35]. High levels of circulating endotoxins are associated with increased organ failure and mortality [36]. The gastrointestinal tract is a major reservoir of bacteria and endotoxins, which can enter systemic circulation if epithelial barriers are compromised [37, 38]. Mammals have evolved mechanisms to tolerate and detoxify endotoxins, and cattle are relatively resistant [39]. LPS administered intravenously at 0.09 μg/kg body weight (BW) elicits an immune response in adult cows [40], while doses of 0.5 and 2.0 μg/kg BW induce endotoxic shock and death in calves, respectively [41, 42].

However, immunocompromised animals and neonatal calves are more susceptible to sepsis [30, 34, 43]. Calves are considerably more susceptible to sepsis than adult cattle, partly because of their immature immune system. Neonatal calves lack prior exposure to pathogens, so their immune systems are naive and respond more slowly to infections. They rely heavily on passive immunity from colostrum, which, if inadequate, leaves them highly exposed to pathogens. Moreover, they have increased gut permeability, not only to absorb immunoglobulins from colostrum in the first days of life, but also because periods of anorexia related to transport or other stressors can heighten the risk of bacterial and endotoxin translocation [43, 44]. Host susceptibility is a more critical determinant of sensitivity to LPS than the direct mechanisms of the endotoxin [45].

This study explores the antimicrobial properties and immune-modulatory effects of five recombinant bovine β-defensins (BNBD1, BNBD2, BNBD3, BNBD4, and TAP) produced in *L. lactis*. We focus on their potential application in treating sepsis by assessing antimicrobial activity against *E. coli*, the capacity to bind LPS and neutralise the induced tumour necrosis factor alpha (TNFα) pro-inflammatory response in whole blood, and the differential stimulation of chemokine IL-8 response in epithelial cells. Here, we present findings on the distinct functionalities of each β-defensin variant, highlighting their possible roles in targeting bacterial pathogens and modulating inflammatory responses.

## Materials and methods

### Recombinant proteins

Five recombinant proteins (Additional file 1) based on the mature sequences of bovine defensins fused to green fluorescent protein (GFP) were used. The proteins BNBD1-GFP-H6, BNBD2-GFP-H6, BNBD3-GFP-H6, BNBD4-GFP-H6, and TAP-GFP-H6 were previously described [46]. GFP-H6 protein was used as a negative control. The encoding genes were cloned into the chloramphenicol-resistant plasmid (Cm^R^) pNZ8148 (MoBiTech) and transformed into the *Lactococcus lactis* strain NZ9000 (pepN::nisRnisK), as described previously [10, 12, 15].

Recombinant protein production and purification were performed as previously described, with minor modifications [47]. Briefly, transformed *L. lactis* strains were grown at 30 °C without agitation in M17 media supplemented with 0.5% glucose and 5 μg/mL Cm for plasmid maintenance. The recombinant proteins were expressed intracellularly under the control of the nisA promoter, induced with nisin (12.5 ng/mL) when the optical density at 600 nm (OD_600_) reached 0.5–0.6. For clarity, these proteins are referred to as rBNBD1, rBNBD2, rBNBD3, rBNBD4, rTAP, and rGFP throughout this paper. Kinetic experiments determined optimal expression times of 3 h for rBNBD2, rBNBD3, and rTAP, and 1 h for rBNBD1 and rBNBD4 (data not shown). Bacterial cells were pelleted at 6200 × *g* for 15 min at 4 °C and stored at −80 °C. Bacterial pellets were resuspended in protein purification binding buffer (20 mM Tris, 500 mM NaCl, 20 mM Imidazole, pH 7.4) and disrupted using a high-pressure homogeniser (Constant Systems CF1 Cell Disrupter); two cycles at 40 kpsi in continuous flow, with samples kept refrigerated.

The soluble fraction was purified by immobilised metal affinity chromatography (IMAC) on HisTrap HP columns with the ÄKTA Start protein purification system (Cytiva) following the protocol in [24]. The eluted fractions were dialysed against 0.01% acetic acid and lyophilised (Telstar LyoQuest lyophiliser) at −60 °C, 0.3 mbar for 18 h. The protein purity was evaluated by SDS–PAGE electrophoresis (TGXTM FastCastTM, Bio-Rad) with Coomassie staining and by western blot. Protein concentration was measured using the Qubit^™^ protein assay kit (Q33211, Invitrogen). Lyophilised protein samples were stored at −20 °C and resuspended before use in 0.01% acetic acid for antimicrobial assays and in 20 mM HEPES prepared with endotoxin-free water for.

### In vitro antimicrobial assay

The bactericidal activity of the recombinant proteins was assessed against *Escherichia coli* BW25113. A single colony was inoculated into 15 mL of Luria–Bertani (LB) broth and incubated for 1 h at 37 °C with agitation at 250 rpm. This culture was subcultured at an initial OD_600_ of 0.05 in 10 mL and grown to an OD_600_ of 0.3–0.4, corresponding to the exponential proliferation phase. The bacterial cells were pelleted at 2500 × *g* for 10 min at 16 °C, washed twice with 0.01% acetic acid, and resuspended to a final concentration equivalent to 10^6^ CFU/mL. Recombinant proteins were mixed with the bacterial cell culture at a 1:1 ratio to achieve a final protein concentration of 1 μM and incubated at 37 °C with gentle agitation for 2 h.

This concentration was selected for all experiments performed because a previous study using recombinant proteins based on defensin sequences demonstrated immunomodulatory activity at 1 μM [48]. A control with protein buffer alone (0.01% acetic acid) was included. Bacterial colony-forming units (CFUs) were enumerated using the drop plate method [49].

Briefly, serial tenfold dilutions in 0.9% (w/v) NaCl were prepared, and 10 μL drops were plated in quintuplicates on lysogeny broth (LB) agar plates. The plates were incubated at 37 °C and the CFUs counted.

### Lipopolysaccharide binding assay

The fluorescent probe BODIPY^®^ cadaverine (BC) (St. Louis, MO, USA) was used to assess the binding of the recombinant proteins to soluble LPS of *E. coli* O111:B4 (437627, Sigma-Aldrich) as previously described [49, 50]. Cadaverine specifically binds to LPS, and when coupled to the fluorophore BODIPY, results in a shift in fluorescence. Thus, LPS binding affinity was evaluated by measuring the displacement of BC at increasing concentrations of the test compound. Recombinant proteins were resuspended in 20 mM HEPES buffer (pH 5.2) and quantified using a Qubit fluorometer (Qubit 4, ThermoFisher). Two-fold serial dilutions of the proteins were prepared in polystyrene 96-well plates using 10 mM HEPES buffer (pH 7.2). LPS and BC were added to achieve final concentrations of 2.5 μg/mL and 2.5 μM, respectively. The final protein concentration ranged from 3 to 0.006 μM. Controls without proteins (NP) or without LPS (NL) were included, as well as a protein scattering control consisting of recombinant proteins at the highest concentration with BC (no LPS). Fluorescence was measured using a TECAN Spark plate reader (Tecan, Männedorf, Switzerland) at 580 nm excitation and 620 nm emission wavelengths, both with a bandwidth of 5 nm, and 30 flashes per well. Protein binding was calculated as:

$$Binding = \frac{Sample - NP}{{NL - NP}}$$

Data were normalised from 0 to 100% to enable comparison among the different proteins, and protein concentrations were transformed to the logarithm. The dose–response curve was fitted to a four-parameter logistic equation to calculate the half-maximal effective concentration (EC_50_): the concentration at which 50% of the protein is bound to LPS.

### Neutralisation effects of LPS in whole blood

The ability of the recombinant proteins to bind to LPS and neutralise their pro-inflammatory effects was assessed ex vivo in bovine whole blood by measuring changes in the secretion of TNFα, a pro-inflammatory cytokine. Blood samples were collected from the jugular veins of 5-month-old calves using EDTA-K2 tubes (BD Vacutainer, Eysins, Switzerland) under a protocol approved by the Animal Care Committee of the Generalitat de Catalunya (project number 12287). Samples were pooled and assayed within 4 h after sampling.

Soluble LPS from *E. coli* 026:B6 (00-4976, eBioscience™, Invitrogen, ThermoFisher) were diluted in phosphate-buffered saline (PBS, pH 7.4) to a concentration of 1 μg/mL and used to induce a substantive increase of TNFα in whole blood. Each recombinant protein (50 μL in 20 mM HEPES, pH 5.2) was mixed with an equal volume of LPS at 0.1 × and 1 × molar concentrations relative to LPS in 1.5 mL sterile Eppendorf tubes. A mixture of the protein buffer (20 mM HEPES) and LPS buffer (PBS) served as a control. After a 30-min incubation at 39 °C, 900 μL of whole blood was added, and samples were then incubated for 2 h at 39 °C with rotation. The serum was collected by centrifugation at 2000 × *g* for 10 min at 4 °C and stored at −80 °C until use. TNFα levels were quantified by ELISA (DY2279, DuoSet, R&D Systems) according to the manufacturer’s instructions. The absorbance was read at 450 nm using a LUMIstar^®^ Omega reader (BMG Labtech).

### Cytotoxicity and regulation of interleukin-8 production in colonic epithelial cells

The cytotoxicity of the recombinant proteins was tested on Caco-2 cells (ATCC HTB-37), a human epithelial cell line derived from colon tissue commonly used in toxicology research. Two independent biological experiments were performed. Caco-2 cells were maintained in Dulbecco’s Modified Eagle Medium (DMEM) supplemented with GlutaMAX^™^ and 1 mM sodium pyruvate (Gibco, Life Technologies) with 10% (v/v) low-endotoxin foetal bovine serum (FBS, A4766801, Gibco, Life Technologies) and 1% penicillin (100 U/mL)/streptomycin (100 μg/mL; HyClone, Thermo Fischer Scientific) in a humidified atmosphere with 5% CO_2_ and at 37 °C. Cells were cultured to 80–90% confluency in 96-well plates and treated with 1, 0.1, and 0.01 μM of the recombinant proteins for 18 h. Cell viability was quantified using an MTT cell proliferation kit (Roche) following standard protocols [49]. A control with cells incubated in protein buffer (20 mM HEPES pH 5.2) was included to represent low cytotoxicity (LC). Before the addition of the MTT reagent, a high cytotoxicity control (HC) was prepared by incubating cells with 1% (v/v) Triton X-100 (T8787, Sigma) for 15 min with 5% CO_2_ at 37 °C. Cell viability was calculated as:

$$Cell viability \left( \% \right) = \frac{sample - HC}{{LC - HC}}x100$$

To determine interleukin-8 (IL-8) secretion, cells were cultured to 80–90% confluency in 24-well plates and treated with 1 μM recombinant proteins for 18 h. We performed two independent biological experiments.

Cell supernatants were collected, centrifuged at 10 000 × *g* for 10 min at 4 °C to remove any debris, and stored at −80 °C. IL-8 levels were quantified using an ELISA kit (Human IL-8, 88-8086, Invitrogen) according to the manufacturer’s protocol, and absorbance was measured with the LUMIstar Omega plate reader (BMG Labtech).

### Statistical analysis

One-way ANOVA with Dunnett’s multiple comparisons test was used to determine the significant differences relative to the control group, while the post-hoc Tukey test was applied to compare all treatments. Results are presented as the mean value ± SEM. Data were analysed using SAS software (version 9.4, SAS Institute Inc.)

## Results

All proteins were successfully produced as GFP-H6 fusion constructs in *L. lactis*, with high purity (≥ 95%), except for rBNBD4, which had slightly lower purity (87%). Production yields varied among the proteins: rBNBD2 and rBNBD3 yielded ~ 2 mg/L; rBNBD4 yielded ~ 1 mg/L; and rBNBD1 and rTAP yielded ~ 0.5 mg/L. The final constructs had molecular weights (MW) of 32–33 KDa and similar hydrophilic characteristics, indicated by a negative GRAVY index (Table 1). All fusion β-defensins exhibited an overall positive charge except rBNBD1, which also had a lower isoelectric point. BNBD1 contains one negatively charged residue and five positively charged residues, insufficient to counterbalance the additional seven negative charges of the carrier protein (GFP), resulting in an overall negative charge (Table 1).

**Table 1:** **Physicochemical parameters of the recombinant proteins**

Protein	MW (Da)	pI	#aa−	#aa +	Hydropathicity
rBNBD1	32549	6.80	35	33	−0.552
rBNBD2	32919	8.34	34	37	−0.586
rBNBD3	33104	8.34	34	37	−0.595
rBNBD4	33053	8.07	34	36	−0.574
rTAP	32389	8.32	34	37	−0.568
rGFP	27468	6.13	34	27	−0.632

Molecular Weight (MW); Isoelectric Point (pI); Negatively- and Positively-charged residues (#aa- and #aa +); hydropathicity defined by the grand average of hydropathicity (GRAVY).

Parameters were calculated using ProtParam [58].

**Figure 1:**
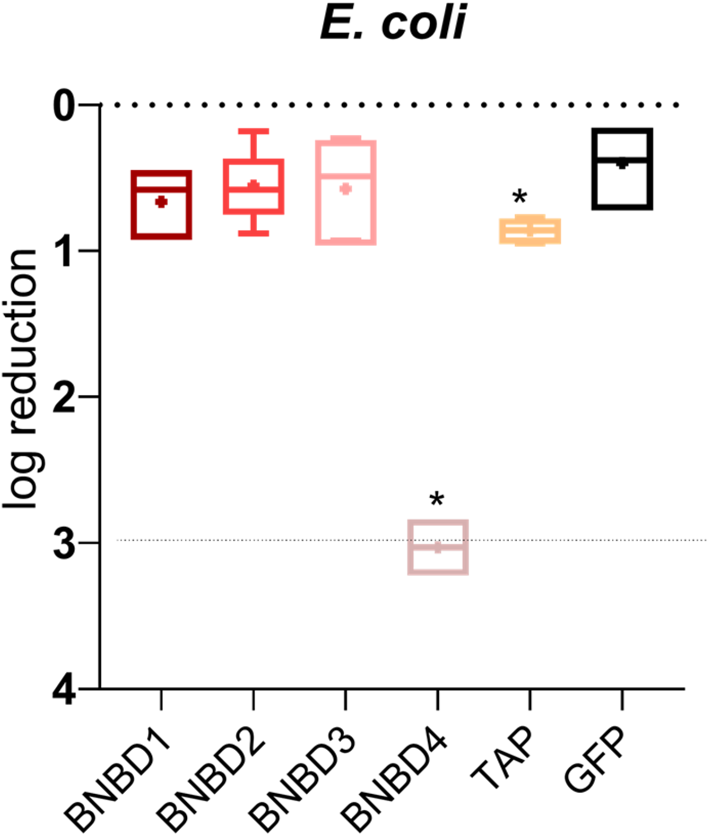
**Antimicrobial activity of recombinant bovine β-defensins against *E. coli*.** The antimicrobial effect was assessed using the drop plate method. The drops were plated in quintuplicates, and the whole process was run in duplicates. Data are shown in a boxplot graph: the whiskers represent the highest and lowest values, and the dots represent the mean value. The detection limit of the assay is a concentration reduction of 3 log_10_ CFU/mL. The statistical analysis was performed as a one-way ANOVA with Dunnett’s multiple comparisons test to compare each protein to the rGFP control; (*) = *p* < 0.05.

The antimicrobial activity of the recombinant β-defensins against *E. coli* was studied. Among the five recombinant β-defensins tested, only rTAP and rBNBD4 exhibited significant antimicrobial activity, with rBNBD4 being the most active, reducing the initial concentration of *E. coli* by ≥ 3 log units (Figure 1). At 1 μM, rBNBD1, rBNBD2, and rBNBD3 did not display antimicrobial activity compared to the control GFP protein.

**Figure 2:**
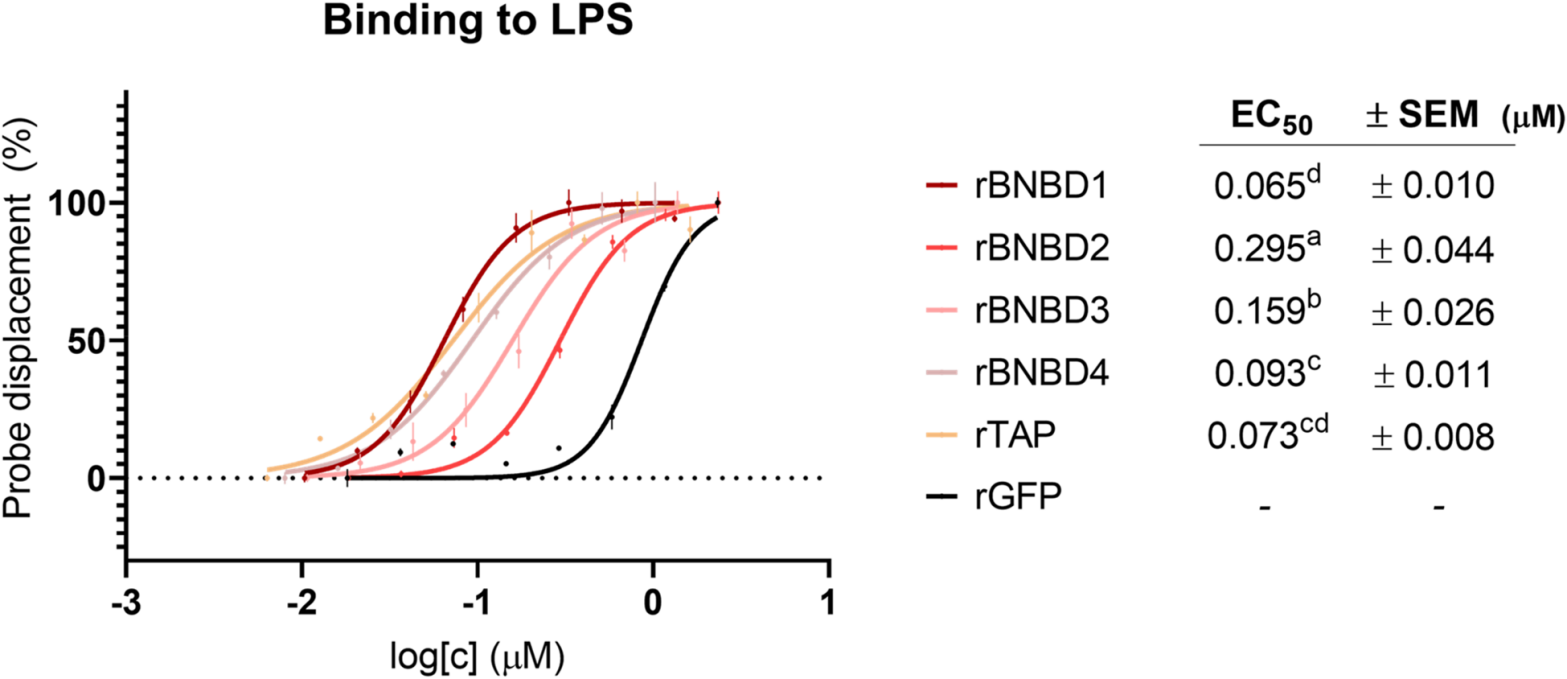
**Binding of the recombinant β-defensins to soluble LPS*.*** The binding was evaluated as the displacement of BIODIPY cadaverine (BC) at increasing concentrations of the recombinant proteins. The data shown is normalised to run from 0 to 100% and fitted to a four-parameter logistic equation to calculate the EC_50_ of each protein. The experiment was run in triplicate, and each data point is the mean value ± SEM. EC_50_ values are shown in concentration units (μM). A one-way ANOVA corrected with Tukey multiple comparisons test was performed on logEC_50_ values, and statistical differences are denoted with letters.

The ability of recombinant β-defensins to bind and neutralise LPS was assessed. In general, all the β-defensins showed LPS binding activity, achieving low EC_50_ values (Figure 2) that ranged from 0.065 to 0.295 μM. Among all recombinant β-defensins tested, the best values were observed for rBNBD1 (EC_50_ = 0.065 ± 0.010 μM), rTAP (EC_50_ = 0.073 ± 0.008 μM), and rBNBD4 (EC_50_ = 0.093 ± 0.011 μM).

**Figure 3:**
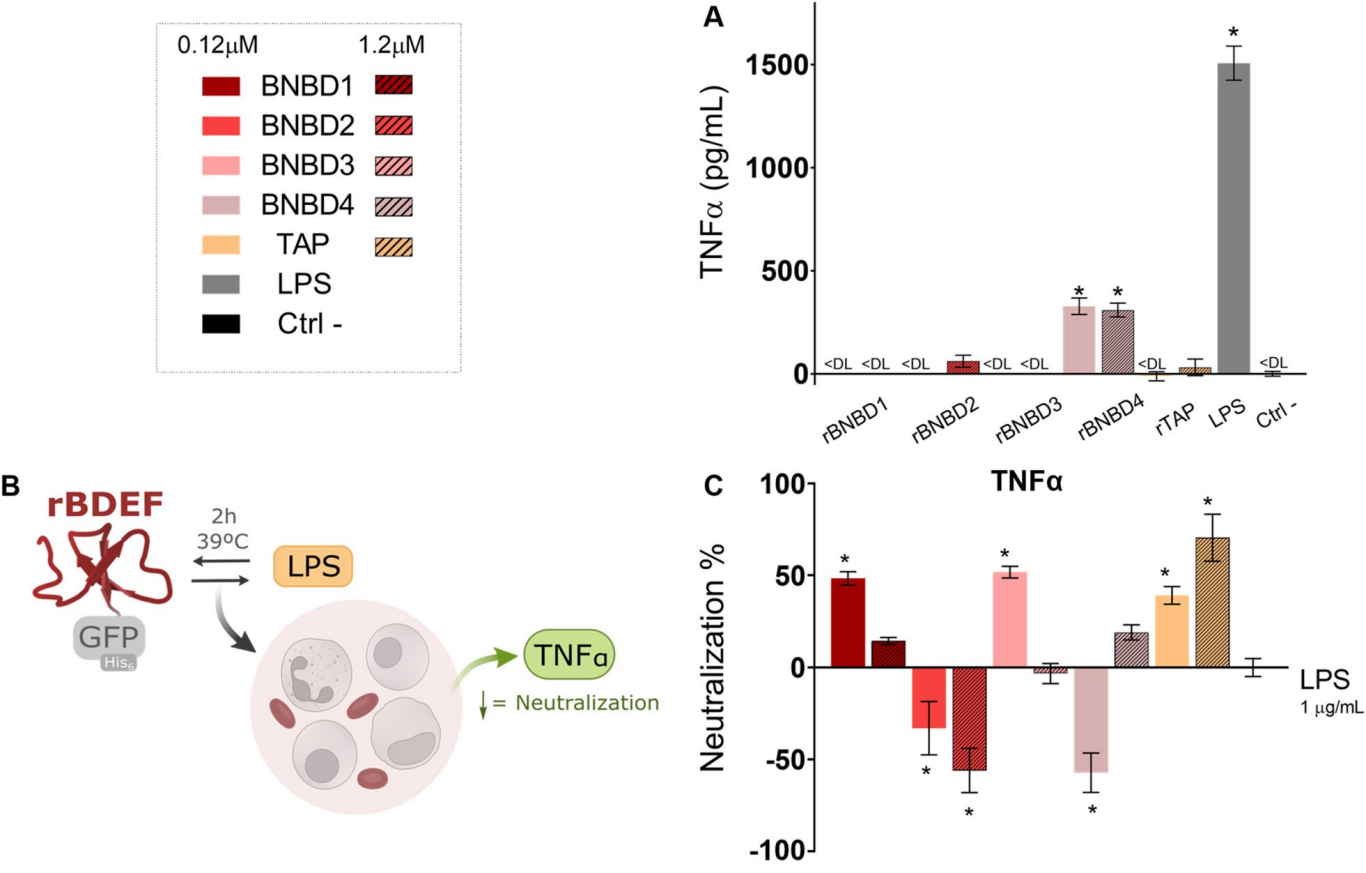
**Neutralisation of the pro-inflammatory effects induced by LPS in whole blood. A** TNFα induced by the recombinant proteins alone at 1 μM or LPS at 1 μg/mL. “ < DL” indicates values under the detection limit of the technique. **B** Representation of the experimental design to test the neutralisation of LPS. **C** The neutralisation ability of the proteins represented in % of the TNFα induced by the proteins with LPS relative to the one induced by LPS alone. The recombinant proteins were incubated with LPS, and the mixture was added to whole blood. Data represented as mean values ± SEM. One-way ANOVA with Dunnett’s multiple comparisons tests was used to determine differences compared to the negative cell control (**A**) or LPS alone (**B**); (*) = *p* < 0.05.

In this context, we also evaluated the effects of the recombinant β-defensins on the secretion of TNFα, a pro-inflammatory cytokine associated with the pathological effects of sepsis [41, 50], induced by LPS in bovine whole blood cells. LPS induced a clear secretion of TNFα, whereas, in general, the recombinant β-defensins did not elevate TNFα levels, except for rBNBD4, which produced low levels of TNFα (Figure 3A). By contrast, rBNBD1, rBNBD3, and rTAP had a clear potential to neutralise the LPS pro-inflammatory response by up to 50% (Figure 3B). However, the rBNBD2 and rBNBD4 had the opposite effect, exacerbating the LPS-driven inflammatory response (Figure 3B).

**Figure 4:**
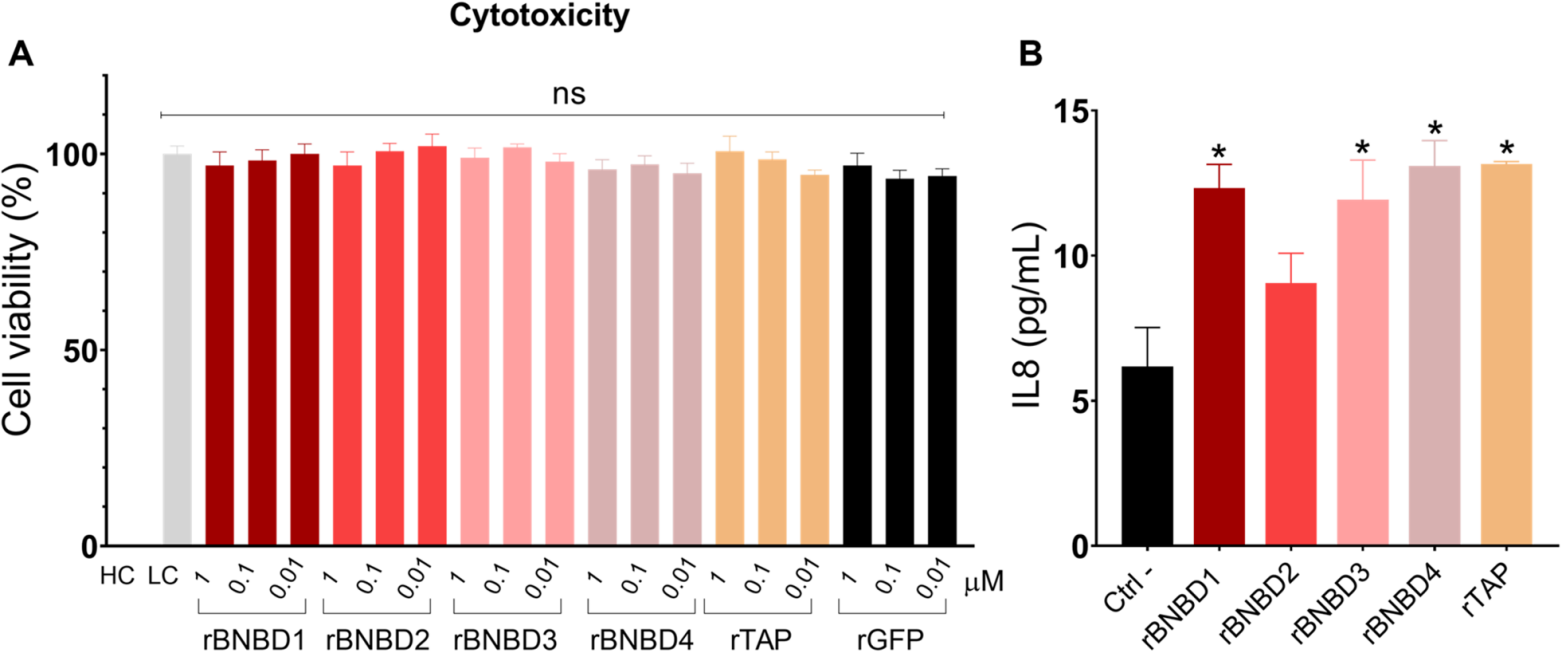
**Effect of the recombinant β-defensins on colonic epithelial cells (Caco-2).** Cells were treated at 80–90% confluence. **A** Cytotoxicity was evaluated as the cell viability when treated with protein concentrations of 1, 0.1 and 0.01 μM. High cytotoxicity control (HC): cells treated with 0.1% Triton X-100; Low cytotoxicity control (LC): cells with the protein buffer. **B** The IL-8 secretion was quantified by ELISA on the supernatants of Caco-2 cells stimulated with 1 μM of the recombinant proteins. Samples were run in quadruplicate, and results are shown as the mean ± SEM. In both cases, one-way ANOVA with Dunnett’s multiple comparisons tests was used to determine differences compared to the cell control; (*) = *p* < 0.05.

Recombinant β-defensins were incubated with intestinal epithelial cells to evaluate their potential toxicity. No toxicity was observed in Caco-2 cells at any of the tested concentrations (0.01 μM, 0.1 μM or 1 μM) (Figure 4A). Following overnight treatment with the HDPs, a small increase in IL-8 secretion was detected for rBNBD1, rBNBD3, rBNBD4, and rTAP (Figure 4B).

## Discussion

The role of bovine defensins in regulating several processes associated with sepsis has been investigated. In this study, we selected five bovine β-defensins: the tracheal antimicrobial peptide and bovine neutrophil β-defensins 1 (BNBD1), 2 (BNBD2), 3 (BNBD3), and 4 (BNBD4). All were previously associated with antimicrobial activity against *E. coli* and *Staphylococcus aureus*, except for BNBD1, which exhibited only moderate activity against *E. coli* [2]. BNBD4 has been linked to defence against bacterial infections of the udder, as its levels increased in animals with mastitis [16]. TAP, meanwhile, has been reported to display broad-spectrum antimicrobial activity (effective against *E. coli*, *Klebsiella pneumoniae*, *Pseudomonas aeruginosa*, *S. aureus* and *Candida albicans*) [1], and its expression is induced by LPS in various epithelial cells [10, 12, 15].

We employed a recombinant strategy to produce the bovine β-defensins by fusing these HDPs to a carrier protein GFP, a design previously used to express other HDPs in *E. coli* [24]. Incorporating a carrier protein provides a minimal additional polypeptide length, reducing susceptibility to proteolysis and neutralising the potential toxic effect of the peptide on the bacterial production host [20, 26, 51].

The production yields were lower than some reported by Aghaei et al., who achieved a higher yield for BNBD2 when produced in *E. coli* (~ 12 mg/L) [22]. This discrepancy may be due to *E. coli* being more extensively studied and optimised as a cell factory, whereas further optimisation is needed for the production in *L. lactis*. Our results are not directly comparable to other studies using *L. lactis*, as some employed the cell lysate as the final product [27] or did not provide data on peptide yield and purity. Nonetheless, we have demonstrated that production in *L. lactis* is feasible without impairing bacterial growth, offering a more economical alternative to standard techniques of chemical synthesis, which are generally not viable for low-cost products required in animal applications.

**Figure 5:**
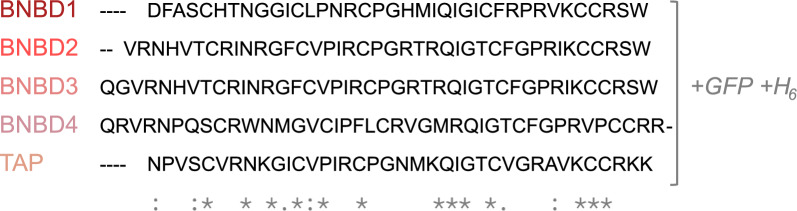
**Sequence alignment of bovine β-defensins BNBD1, 2, 3 and 4, and TAP using Clustal Omega.** (*) indicates a fully conserved residue; (:) indicates conservation of residues with strongly similar properties; (.) indicates conservation of residues with weakly similar properties.

In this study, rTAP and rBNBD4 showed notable antimicrobial performance (Figure 1), with rBNBD4 being the most effective. The antimicrobial activity of these β-defensins isolated from their natural sources against *E. coli* has been previously reported [1, 2]; however, differences in experimental techniques preclude direct comparison. Selsted et al. found that among the neutrophil β-defensins, BNBD4 exhibited the strongest antibacterial activity and BNBD1 the weakest [2]. Aghaei et al. observed that recombinant BNBD2 cleaved from its carrier protein was active against *E. coli* and *S. aureus* at 0.05, 0.1, and 0.2 mg/mL (~ 11, 22, 43 μM), but was inactive in its fusion protein form (BNBD2 fused to thioredoxin and a His-tag) [22]. In terms of amino acid composition, all the β-defensins tested contained similar numbers of negatively and positively charged residues and exhibited comparable hydrophobicity values (Table 1). However, in BNBD4, the distribution of positively charged arginine (R) residues differed, with two consecutive arginines and two additional closely positioned arginines at the C- and N-termini, respectively (Figure 5), which could potentially account for the increased antimicrobial activity of this defensin. This finding is consistent with the observations of Klüver et al. [19], who demonstrated that antimicrobial activity is influenced by the distribution of positively charged amino acids and hydrophobic side chains.

All recombinant β-defensins bound LPS efficiently compared to the GFP control, indicating that their binding affinities were specific to the β-defensins. The strongest binding was observed for rBNBD1, rTAP and rBNBD4. These results also showed that LPS binding is not necessarily correlated with antimicrobial activity, as rBNBD1 bound efficiently to LPS but did not exhibit bactericidal activity against *E. coli* (Figure 1). Similar to other HDPs, the direct antimicrobial activity of β-defensins depends on their cationic and amphipathic nature: the peptides are attracted to negatively charged membranes, which they can then penetrate to induce membrane depolarisation or disruption [52, 53].

The overall negative charge of the designed rBNBD1 could impair the ability of BNBD1 to interact with *E. coli*, thereby reducing its bactericidal effectiveness. As LPS can be released from the bacterial membrane during cell proliferation and death, direct binding to soluble LPS is also crucial in reducing immune cells and preventing a dysregulated immune response.

Our study of LPS neutralisation indicated that binding alone might not be the sole parameter governing the neutralisation of the pro-inflammatory effects. rBNBD4 bound LPS efficiently (Figure 2) but did not neutralise the secretion of TNFα induced by the endotoxin (Figure 3B). Clinically, this suggests that some defensins may act as LPS scavengers without mitigating downstream pro-inflammatory effects, thereby limiting their standalone therapeutic potential in sepsis. By contrast, rBNBD3 did not exhibit a particularly strong binding capacity (Figure 2), yet it demonstrated neutralisation activity (Figure 3B). Although some β-defensins, such as human DEFB123, DEFB114, and DEFB118, have been shown to bind and neutralise LPS [4, 7, 54], other studies indicate that these activities are not always directly linked. For example, human β-defensin 3 does not bind to LPS but can reduce the levels of LPS-induced TNFα in vitro and in vivo [55]. It is suggested that this β-defensin regulates the secretion of TNFα by affecting signalling molecules downstream of the TLR4 activated by LPS.

It is also noteworthy that rBNBD2 and rBNBD4 increased the pro-inflammatory response to LPS (Figure 3B). While this is undesirable in the context of sepsis, it could be valuable in other applications, such as their use as adjuvants.

Epithelial cells in barrier tissues play a crucial role in immune defence against infections by detecting, signalling, and helping eliminate external pathogens. This includes recruiting immune cells to infection sites, for example, by secreting the chemokine interleukin-8 (IL-8) in response to pathogen-associated molecular patterns (PAMPs).

The role of IL-8 in mucosal immunity has been well established, as it acts as a potent chemoattractant for neutrophils and an initiator of inflammation [56]. Prolonged exposure to IL-8 can lead to chronic inflammation, and elevated IL-8 levels are associated with increased mortality in septic patients [57]. However, none of the β-defensins studied induced high levels of this cytokine, thereby reducing the risk of exacerbating inflammation while still slightly activating mucosal immunity, which could be beneficial in halting an infection.

Overall, our results showed that β-defensins exhibit a wide range of activities relevant to *E. coli* infections and sepsis, suggesting potential therapeutic use in cattle (Table 2). None of the recombinant defensins tested combined all activities simultaneously, highlighting the multifunctional role and the mechanistic diversity of these peptides. Generally, rTAP demonstrated antimicrobial effects against *E. coli* alongside a broader spectrum of immune-regulating activities: binding and neutralising LPS, and activating mucosal immunity. The combination of these activities could support sepsis treatment by reducing bacterial load while neutralising the LPS pro-inflammatory effects. In contrast, rBNBD4 showed a stronger antimicrobial activity.

**Table 2:** **Summary of results**

Protein	Antimicrobial activity (*E. coli*)	LPS binding	Cytotox (Epithelia)	Cytokine secretion		LPS neutralisation (Whole blood)
				IL-8 (Epithelial cells)	TNFα (Whole blood)	
rBNBD1	−	+ + + +	−	+	−	+
rBNBD2	−	+	−	−	−	−
rBNBD3	−	+ +	−	+	−	+
rBNBD4	+ + +	+ + +	−	+	+	−
rTAP	+	+ + + +	−	+	−	+ +

For each experiment, a “ ± “ designation was assigned using arbitrary criteria. Antimicrobial activity: ( +) = 1 log difference; (−) = 0 log difference. LPS binding: statistical differences denoted letter* a* = ( +); *b* = (+ +); *c* = (+ + +); d = (+ +  + +). Cytotoxicity: not significant = (−). Cytokine secretion (IL-8 and TNFα): not significant = (−) and statistical differences ( +). LPS neutralisation (for each concentration of protein): neutralisation (decrease TNFα) = ( +); no neutralisation (increase of TNFα) = (−), not significant = blank.

Future studies should evaluate whether the activity of β-defensins can be combined in a fusion protein with TAP and BNBD4 alone, without the need for a carrier protein. The primary target in cattle would be neonatal calves, where sepsis remains a leading cause of mortality. This will require assessing effects across multiple doses to define the optimal therapeutic window, the protein’s half-life and degradation profile under physiological conditions, and any potential to elicit adverse immune responses, thereby providing a clearer roadmap for preclinical and clinical development.

## Supplementary Information

**Additional file 1. Protein sequences.**

## Data Availability

All data generated or analysed during this study are included in this published article [and its supplementary information files].
