# Supplementary material for: Exploring the therapeutic potential of recombinant bovine β-defensins for antimicrobial and anti-inflammatory functions in sepsis management
Source: Vet Res. 2025 Sep 2;56:173. doi: 10.1186/s13567-025-01601-0 (PMC12403256; doi:10.1186/s13567-025-01601-0)
Supplement: Supplementary file 1 — Additional file 1. Protein sequences. [file 13567_2025_1601_MOESM1_ESM.docx]

**Additional file 1. Protein sequences.**

Complete protein sequences. GFP sequence in italics, β-defensins sequences in bold, linker underlined and His-tag in subindex.
